# Distribution characteristics and ecological risk assessment of heavy metals in sediments of Shahe reservoir

**DOI:** 10.1038/s41598-022-20540-w

**Published:** 2022-09-28

**Authors:** Wen Sun, Ke Yang, Risheng Li, Tianqing Chen, Longfei Xia, Xubo Sun, Zhao Wang

**Affiliations:** 1 Shaanxi Provincial Land Engineering Construction Group, Key Laboratory of Degraded and Unused Land Consolidation Engineering, Ministry of Natural Resources, Xian, 710075 China; 2 Shaanxi Provincial Land Engineering Construction Group, Shaanxi Provincial Land Consolidation Engineering Technology Research Center, Xian, 710075 China; 3 Shaanxi Provincial Land Engineering Construction Group, Land Engineering Technology Innovation Center, Ministry of Natural Resources, Xian, 710075 China; 4 Land Engineering Quality Testing of Shaanxi Land Engineering Construction Group Co., Ltd, Xian, 710075 China

**Keywords:** Environmental sciences, Environmental social sciences

## Abstract

Shahe Reservoir of Northern Canal Basin was selected as the study area. Nineteen surface (0~20 cm) sediment samples and three sediment core samples were collected to analyze the spatial distribution characteristics of As, Cd, Cu, Mn, Pb and Zn in the sediments. The geo-accumulation index, potential ecological risk index and risk assessment code were used to evaluate heavy metal pollution, as well as its potential risk was analyzed according to the speciation of heavy metals. Results showed that the average enrichment factors of heavy metals compared to the background value in soil of Beijing were ranked at the order as the point source pollution area > the central area of the reservoir > the downstream area of the reservoir > the Nansha River > the upstream area of the reservoir > the Beisha River, namely, 2.57 times, 2.06 times, 1.97 times, 1.95 times, 1.87 times and 1.85 times, respectively. The sediment core samples in the central area of the reservoir and the inlet of the Beisha River showed a trend of increasing firstly and then decreasing with the change of depth. Pollution assessment results showed that sediment was moderately contaminated or moderately to strongly contaminated by As, but the other heavy metals were not polluted or lightly polluted. The potential ecological risk index of all sampling sites was less than 150, showing a low ecological risk. As Cr and Cu were mainly in the speciation of residues, with low bioavailability. Although the content of Mn and Zn were low, they showed high bioavailability. Based on correlation analysis and principal component analysis, it was speculated that the sources of various heavy metal pollution in the sediment were similar, which were possibly input from the external wastewater. The heavy metals in sediment were positively related to nutrients and organic matter, indicating that all of them were mostly from the same point polluted sources.

## Introduction

Heavy metal (HM) environmental pollution is one of the consequences of economic development in human societies^1^. The anthropogenic HM enrichment of the environment can be a result of industrial development, transport, agriculture, and settlement activities, posing a serious threat to the downstream aquatic ecosystems^2^. Several studies widely reported the obvious bioaccumulation and biomagnification properties of HMs in reservoir sediments and food webs^3,4^. Under ideal conditions, HMs in the sediments can be re-released through various physical and chemical processes such as precipitation and dissolution, oxidation and reduction, adsorption and desorption, causing secondary water pollution. Therefore, the widespread concern of HM pollution in sediments has become an extensive research domain. Analysis of the spatial distribution, pollution evaluation, morphological characteristics, potential sources and the interannual changes collectively shows the pervasiveness of HMs in sediments within typical rivers, lakes and reservoirs such as Taihu Lake, Chaohu Lake, Miyun reservoir, Dianchi Lake, and Caohai Lake in China^5–8^.

As the basin with highly concentrated population, densest industries and the booming pace of urbanization in Beijing, the North Canal Basin has traditionally been tasked for flood control, irrigation, sewage discharge and other functions. This watershed is also characterized with a concentrated local population, and undertakes 90% of the drainage services in the central urban area of Beijing. The effluent discharge from diverse industries potentiates pollutant deposit in the river sediments, which has been substantially inclined in recent years^9^. A previous study reported abundant HM content in the surface sediments of the Wenyu reach of the North Canal^10^.The content of HMs in the sediments of the upstream urban reaches is higher than that of the downstream rural reaches, and the content of some heavy metals shows a trend of increasing from the bottom of the sediment to the surface^11^. Importantly, considerable enrichment of HMs contents was observed in the middle of North Canal at Shahe reservoir relative to those found in the outlet and inlet points. The commonly reported HMs include Cr, Cu, and Zn sourced from anthropogenic inputs, while Mn and Pb inflows are pervasive by natural sources^12^. However, systematic knowledge on the spatial distribution aspects, ecological risk assessment, and the source analysis of HMs within the sediments of Shahe reservoir in the headwaters of the North Canal remains largely unmapped.

Therefore, this study collected 19 surface sediment samples and 3 sediment column samples from the Shahe reservoir, and analyzed the spatial distribution characteristics of six widely documented HMs. Besides, The SPSS 25.0 sofware was used to analyze the principal component of HMs with N and P pollutants in sediments.The spatial distribution and the percent content of various speciation of heavy metals were analyzed by Origin 2017.This study is projected to disclose the drivers of HM pollution in the Shahe reservoir and provided a theoretical basis for the research and prevention of heavy metal pollution in Shahe Reservoir and its similar channel-type reservoirs.

## Materials and methods

### Brief mapping of the studied area

The North Canal water system originates from the Southern foot of Yanshan Mountain in Changping District, Beijing, and flows through Beijing, Langfang, Hebei Province and Tianjin, successively. The Wenyu river runs above the Beiguan gate i.e., the city’s sub-center, and the North Canal flows below the Beiguan gate. All the way, it flows alongside three plain rivers including the Tonghui river, Liangshui river, and the Fenggang Jianhe river, and it joins the Haihe river after the Qujiadian and the Yongding river convergence^13^. Shahe reservoir is a channel-style reservoir in the basin of the North Canal and was built in 1960. The catchment mainly originates from the Shahe reservoir basin in Changping, Yanqing and Haidian. Both the Dongsha and the Wenyu river in the lower outreach are the key junctions in the upper reach of the North Canal^14,15^. The reservoir spans about 1.8 km^2^, with an approximate 20.45 million m^3^ storage capacity, and the rainwater and sewage constitutes the major share of the incoming water^12^. The sediments of the Shahe Reservoir are mainly mud accompanied by a small number of dead branches and leaves washed by the upstream water.Sandy loam is the main covering soil in the Shahe Reservoir area, and the land use in the area is mainly construction land such as schools and communities.

### Sample collection and analysis

**Figure 1:**
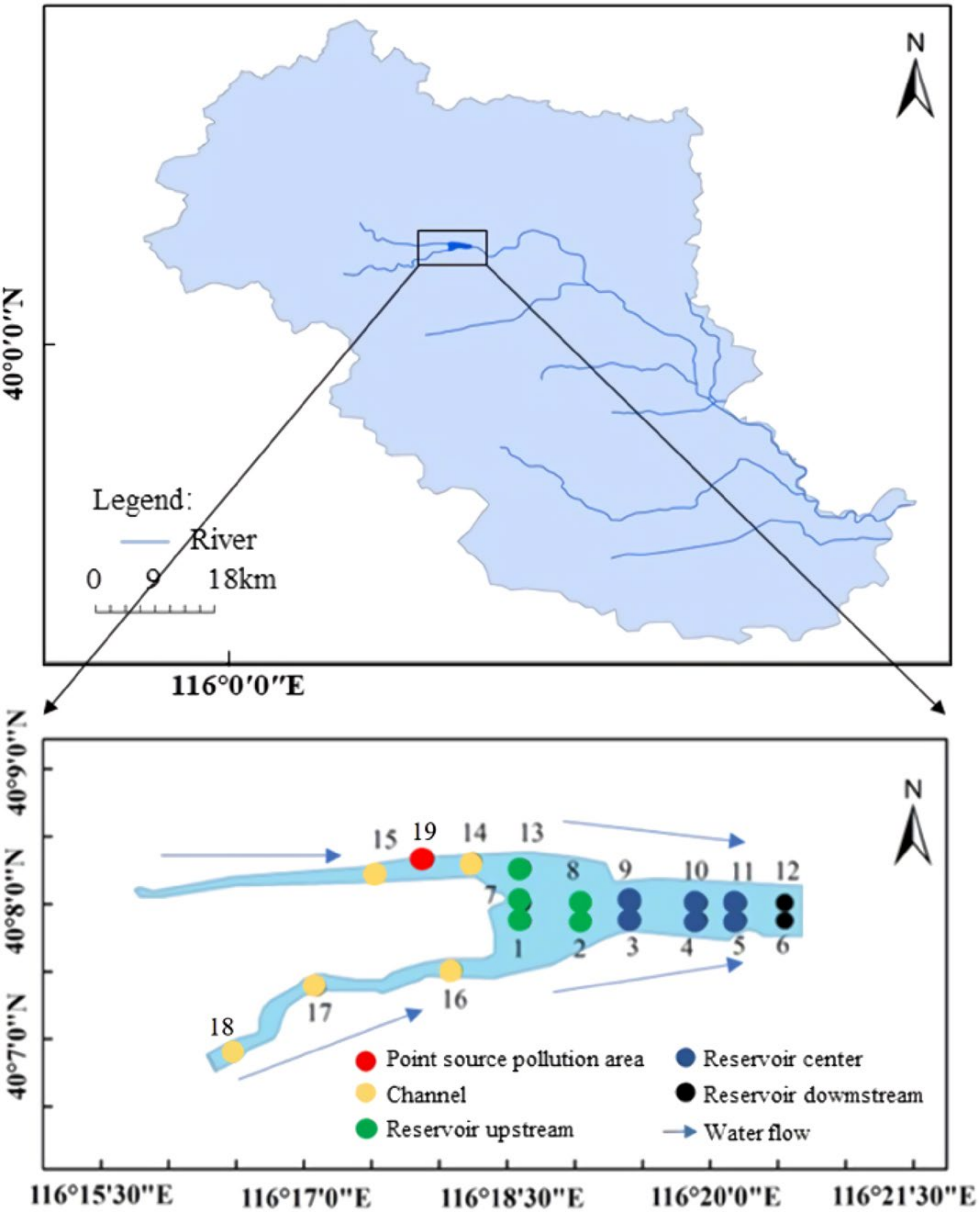
Arrangement and zoning map of sediment sampling points in Shahe Reservoir. (The figure was created by Wen^16^ and modifed using ArcGIS sofware 10.2; Source: WGS 1984).

A total of 18 sampling points were set up in the Shahe reservoir in Nov. 2017 and the distribution of sampling points has shown in Fig. 1. Specifically, these spots were positioned in the upper reaches (1^#^, 2^#^, 7^#^, 8^#^, 13^#^), the central area (3^#^, 4^#^, 5^#^, 9^#^, 10^#^, 11^#^) downstream site (6^#^, 12^#^), Channel (14^#^, 15^#^, 16^#^, 17^#^, 18^#^), and the point source pollution area (19^#^). The sediment samples at the surface (0–20 cm) were collected by the Peterson sampler, and the self-gravity columnar mud harvester was used to fetch three sediment columnar samples at points 3^#^ (0–42 cm), 14^#^(0–30 cm) and 16^#^(0–26 cm). The collected sediment columnar samples were layered by 2 cm. Afterwards, the layered samples and the surface sediment samples were freeze-dried (FD-1A-50 freeze dryer, Beijing Boyikang Experimental Instrument Co., Ltd.), followed by the removal of gravel and the other fragments. The plant residues and the other impurities were ground and passed through a 100-mesh sieve. The collected sediment samples were from the similar batch as those analyzed by Sun et al., that investigated the nutrient and organic matter (OM) content of targeted sediments^16^.

The obtained sediment samples were refined by the Tessier method with minor modifications^17^. Take 0.5000*g* of the sediment sample filtered with a 0.149 mm sieve after freeze-drying, add 10 mL of HNO_3_, 1 mL of HClO_4_, 1 mL of HF, digest with microwave(120 °C, 1 h–160 °C, 1 h–180 °C, 0.5 h), and finally make up to 50 mL with ultrapure water (Milli-Q Advantage A10, Merck Millipore,USA) then followed by the determination of As, Cr, Cu, Mn, Pb, and Zn content by Inductively Coupled Plasma Optical Emission Spectrometer (ICP-OES) (OPTIMA 2000DV, Perkin Elmer, USA)^18^. In the analysis process, the program blank and standard material (GBW07301, GBW07304, GBW07308, Institute of Geophysical and Geochemical Exploration, Chinese Academy of Geological Sciences) for the analysis of water system sediment components are used to control the accuracy of the analysis.The speciation of HMs in sediment samples was analyzed by an improved Community Bureau of Reference (BCR) continuous fractional extraction method^19^. Moreover, the entire HM speciation scheme was discussed in four forms namely; F1 (acid-extractable state), F2 (reducible state), F3 (oxidizable state), F4 (residue state), with subsequent ICP-OES analysis to determine the individual HM content of each form of HMs in the sample extracts.

### Pollution assessment methods

#### Index of geoaccumulation method

The index of geoaccumulation method was used in this study^20^ to rate HM pollution in sediments with below stated formula:

1 $${I}_{geo}={log}_{2}\left[\frac{{C}^{i}}{K\times {B}_{i}}\right]$$

where *C*^*i*^ was the measured concentration for ith HMs (mg kg^−1^); *B*_*i*_ was the environmental background value of the measured element. Importantly, the background value of soil elements in Beijing was taken in compliance with the evaluation benchmark (Table 4)^10,21^, *K* was a constant with typical score of 1.5. According to the calculated value of the geo-accumulation index *I*_*geo*_, the degree of HM pollution can generally be divided into seven grades as shown in Table 1.

**Table 1:** Values of *I*_*geo*_ and the pollution level.

*I*_*geo*_	Grading	Degree of pollution
< 0	0	No pollution
0~1	1	Slight pollution
1~2	2	Near to moderate pollution
2~3	3	Moderate pollution
3~4	4	Near to heavy pollution
4~5	5	Heavy pollution
> 5	6	Serious pollution

#### Potential ecological risk index method

The proposed potential ecological risk index is a fast, simple and standard method and is based on the sedimentology principles to model the potential ecological risk of HMs^22^ with following Eq. (2):

2 $$RI=\sum_{i=1}^{m}{E}_{r}^{i}=\sum_{i=1}^{m}\left[{T}_{r}^{i}\cdot \left({C}^{i}/{C}_{n}^{i}\right)\right]$$

Here, the ${E}_{r}^{i}$ was the potential ecological hazard coefficient of the ith heavy metal; ${T}_{r}^{i}$ was the toxicity response coefficient for the ith HM, reflecting its toxicity level and corresponding sensitivity to the pervasive pollution source (HM). The designated toxicity response coefficients for As, Cr, Cu, Mn, Pb, and Zn were 10, 2, 5, 1, 5, and 1, respectively^23^; ${C}^{i}$ was the measured concentration of the ith HM (mg kg^−1^); ${C}_{n}^{i}$ was the evaluation reference value for ith HM (Table 4). According to the ${E}_{r}^{i}$ and $RI$ values, the potential ecological risks of soil HMs can be classified with the following scale (Table 2).

**Table 2:** Classification of potential ecological risk factor.

Potential ecological hazard factor (${E}_{r}^{i}$)	Potential ecological risk index(*RI*)	Potential ecological risk level classification
< 40	< 150	Mild ecological hazard
40~80	150~300	Moderate ecological hazard
80~160	300~600	Strong ecological hazard
160~320	> 600	Intensity ecological hazard
> 320	/	Extremely severe ecological hazard

#### Risk assessment coding

The risk assessment coding (RAC) method was proposed. to evaluate the bioavailability of HMs in the sediments based on the exchangeable fraction (F1) content (Eq. 3)^24^.

3 $$RAC=\frac{{C}_{F1}}{{C}_{tot}}\times 100\%$$

Here, the ${C}_{F1}$ was the acid extractable state content (F1); ${C}_{tot}$ was the total HM content. The model RAC classification has shown in Table 3.

**Table 3:** Classification of risk assessment code.

RAC	Grading	Risk level
< 1%	1	No risk
1–10%	2	Low risk
10–30%	3	Medium risk
30–50%	4	High risk
> 50%	5	Extremely high risk

### Data processing and analysis methods

The spatial distribution characteristics of HMs in sediments were analyzed by OriginPro 2017 software. Pearson correlation analysis was performed with SPSS 26.0 software, and PCA was performed with Canoca 5.0.

## Results and discussion

### Spatial distribution characteristics of HMs in sediments

As stated earlier, the 19 sampling points were classified into 6 subgroups; the upper reaches, central area, the lower zone, Beisha river, Nansha river and the point source pollution area (Table 4). The results indicated a differential accumulation pattern for six HMs in the sediments of each zone of the Shahe reservoir.The average content of As, Cr, Cu and Zn in surface sediments was 5.04-, 1.96-, 2.17- and 2.19-times higher than that in the soil background value in Beijing respectively. Among all figures, the average content of As was (35.85 ± 5.80) mg kg^−1^, which was seriously higher than the data in Beijing. According to the study of Hu et al.^25^, the soil background value of As in Wenyu River section of North Canal was 22.71 mg kg^−1^, which is 3.2 times higher than that in the soil background value in Beijing. Hu’s study demonstrates the background value of As in surface sediments is higher in Wenyu River per se, and the reason behind is possibly the location of North Canal. As North Canal lies in semi-rural area and is largely occupied by agroforestry, the herbicides and pesticides applied in agriculture can bring about serious As pollution.

**Table 4:** HM content in surface sediments and soil background level of Beijing mg kg^−1^.

Sampling site		As	Cr	Cu	Mn	Pb	Zn
Upper reservoir 1^#^, 2^#^, 7^#^, 8^#^, 13^#^	Content	30.79~39.60	33.66~76.52	16.22~46.98	307.52~568.41	15.70~29.91	30.67~273.08
	Mean	34.35 ± 3.43	55.03 ± 16.14	32.84 ± 11.78	406.22 ± 110.32	22.95 ± 5.27	115.76 ± 96.27
Reservoir Center Area 3^#^, 4^#^, 5^#^, 9^#^, 10^#^, 11^#^	Content	27.64~43.03	43.52~81.60	32.09~57.29	102.95~668.18	10.72~36.41	16.65~173.31
	Mean	35.88 ± 5.82	62.24 ± 13.33	45.84 ± 8.59	369.44 ± 212.13	26.45 ± 10.40	98.90 ± 64.92
Downstream of the Reservoir 6^#^, 12^#^	Mean	33.86 ± 4.34	57.31 ± 1.70	39.59 ± 1.80	342.82 ± 3.45	27.54 ± 2.57	107.39 ± 22.98
Beisha river 14^#^, 15^#^	Mean	32.99 ± 3.19	55.31 ± 7.01	34.69 ± 14.82	344.10 ± 132.82	23.37 ± 8.19	113.71 ± 51.09
Nansha river 16^#^, 17^#^	Mean	35.14 ± 0.0.58	60.87 ± 3.05	35.64 ± 0.96	255.06 ± 52.23	16.62 ± 10.96	141.58 ± 72.05
Point source pollution Area 18^#^, 19^#^	Mean	44.34 ± 13.35	56.01 ± 5.80	56.64 ± 10.87	276.94 ± 33.25	28.99 ± 5.21	246.14 ± 46.32
Reservoir sediment mean		35.75 ± 5.80	58.29 ± 11.09	40.65 ± 11.47	351.87 ± 137.85	24.45 ± 7.79	125.78 ± 75.97
Beijing soil background value		7.09	29.80	18.70	571.00	24.60	57.50

**Figure 2:**
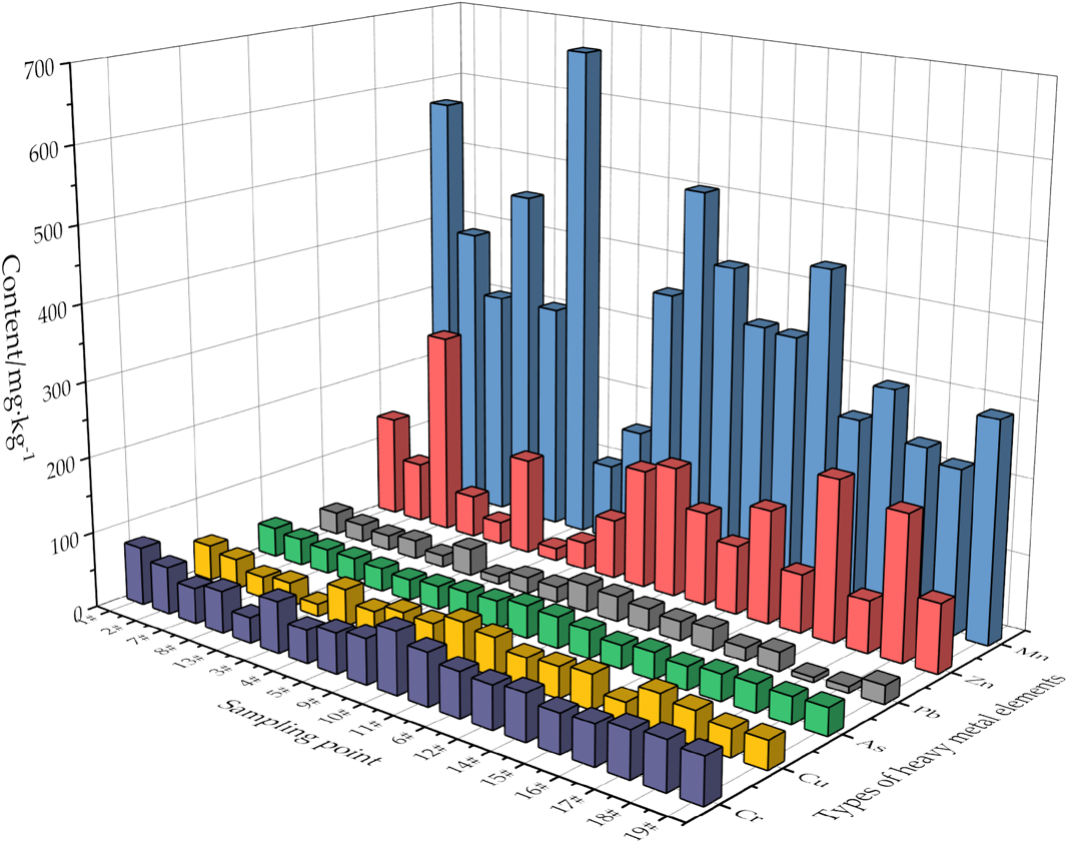
Horizontal distribution characteristics of HMs in sediments.

An obvious difference in the degree of HM pollution was observed in the six investigated regions. The average order of different HM enrichment times in the six regions is as follows: point source pollution area (2.57 times) > reservoir center (2.06 times) > downstream of the reservoir (1.97 times) > Nansha river (1.95 times) > upstream of the reservoir (1.87 times) > Beisha river (1.85 times). As presented in Fig. 2, the horizontal distribution of HMs, at the point source pollution site and the reservoir center was significantly higher compared to the other regions. Notably, within the most seriously polluted point source pollution area, the average content of Mn was lower than the soil background levels. However, the observed average content of As, Cr, Cu, Pb, and Zn was 6.25, 1.88 and 3 times the background value, respectively 3.03, 1.18, 4.28. This could be explained that the Shahe reservoir is located near the urban–rural fringe, and the principal contributors to the point-source pollution site are domestic sewage, surface runoff and confluence system overflow pollution^26^. These results were also correspondent to a previous study demonstrating that urban domestic sewage contains As, Cd, Cu, Cr, Hg, Ni, Pb, Zn and other HMs^27^. In addition, the road surface runoff pollution in Beijing urban region is also serious, and the surface rainfall runoff contains miscellaneous HMs such as Zn, Pb, Cu, Hg etc., which are predominately released by the atmospheric dry and wet deposition as well as by the automobile exhaust^28,29^. Similar results were reported by Chang that different HMs such as Cu, Zn, Pb, and Cd were enriched in pipeline sediments^30^. Therefore, this depicts the augmented HM pollution at the point source pollution site. It is noteworthy that the average content of As, Cr, and Cu in the midpoint was higher than that in the upstream and downstream of the reservoir. This corresponded to slow water flow and poor hydrodynamic conditions in the center of the reservoir. These factors collectively contribute to higher deposition of particulate matter, and the strong adsorption of particulate matter is attributed to different HM species. Except for Mn and Pb, the average contents of six HMs in the Nansha river and the Beisha river were considerably higher compared with the soil background levels in Beijing. More specifically, the average contents of the other five HMs were higher in the Nansha river relative to those in the Beisha river.

**Figure 3:**
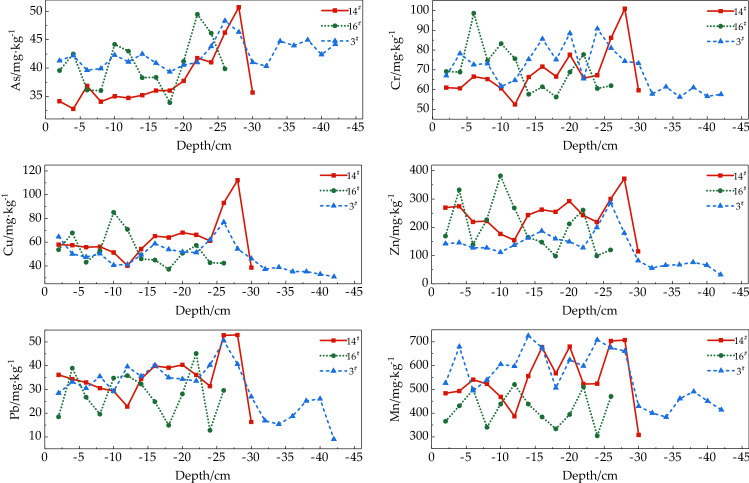
Vertical distribution characteristics of HMs in sediments.

In particular, we discuss the 3^#^ sedimentary column (0–42 cm) in the center area of the Shahe reservoir, the 14^#^ sedimentary column (0–30 cm) at the entrance of the Beisha river, and the 16^#^ sedimentary column (0–26 cm) at the entrance of the Nansha river (see Fig. 3), respectively, as follows. According to the sediment analysis, highest average contents were observed for As, Cr and Mn i.e., 42.42, 70.37, 554.33 mg kg^−1^ respectively, in the vertical direction at the center of reservoir. Compared with the Beijing soil background levels, the proportion of As and Cr was 5.98 and 2.36 times higher respectively, with exclusion of Mn due to no accumulation. At the Beisha river inlet in the vertical direction, highest Cu, Pb, and Zn levels (62.77-, 35.30-, and 240.95- mg kg^−1^, respectively) were observed. Furthermore, this was higher than those in the reservoir center and the Nansha river inlet which were only 3.36, 1.43, and 4.19 times respectively, higher to the soil background levels of Beijing. Notably, the distribution characteristics of the sedimentary columns in the reservoir center (3^#^) and the Beish inlet (14^#^) in the vertical direction were largely similar, with minor variation between the As and Cu contents. In particular, a simultaneous up and down trend for (As and Cu) was observed at the depth of 20–30 cm. This was consistent with the previous literature^12^ that the sediment depths of 0–10-, 10–20-, and 20–30 cm in the Shahe reservoir corresponded to chronological relationships in the typical timeframe of 2000, 1980–2000 and 1960–1980, respectively. It should be noted that the accumulated HM factions were evidently observed in the sediments of the reservoir center and Beisha river inlet from 1960 to 1980 with subsequent gradual decline^12^. After 1980 however, no significant variations were detected in the HM content of sediments over time. Similarly, the sedimentary columns in the reservoir center area (3^#^) and the Beisha inlet (14^#^) tended to increase at first followed by a depth-associated decline in the vertical direction, and the severe HM pollution was predominant at the depth of 25–30 cm. The contents of six HMs exhibited a wave-like scheme in the vertical direction within the sedimentary column of the Nansha river inlet (16^#^). Namely, the Cr content was prominent at 6 cm-, Cu and Zn were at 10 cm-, and the As and Pb contents were observed at a 22 cm depth. Moreover, no obvious interannual variation was observed at the highest level, and the HM contents within the sediments were relatively stable over time.

### Evaluation of HM pollution in sediments

The geo-accumulation index method and potential ecological risk index method were used to evaluate the HM pollution in the surface sediments of Shahe reservoir. Table 5 presents the inspected outcomes of the geo-accumulation index (*I*_*geo*_), single potential ecological risk coefficient (${E}_{r}^{i}$) and the potential ecological risk index (*RI*) of HMs. The results from geo-accumulation index method revealed that except for the 10^#^ site at the central area and the 18^#^ site at the point source pollution area, As has an *I*_*geo*_ value of 2~3, suggesting moderate pollution. For the remaining 17 sampling points consistently, the surveyed score ranked for *I*_*geo*_ was 1~2, denoting similar moderate pollution. In this study, the *I*_*geo*_ of Cr and Cu ranged from 0 to 1, which attributed to slightly polluted stage. Among the 19 sampling points for Zn, 7 points were classified as slightly polluted, 5 points were moderately polluted, and the remaining 7 points were notably marked as not polluted. Alternatively, the *I*_*geo*_ value of Mn and Pb points were less than 0 at all sampling, and was classed as a pollution-free state. Hence, the calculated pollution spread of six HMs by the geoaccumulation index method was in the order of As > Cu > Cr > Zn > Pb > Mn. Besides, the applied potential ecological risk index method revealed that the single potential ecological risk coefficient (${E}_{r}^{i}$) of As was 40–80 in 18 sampling points except 3^#^, indicating moderate pollution with lower ecological hazard. Overall, the ${E}_{r}^{i}$ of the remaining six HMs was below 40, which also falls in mild ecological hazard group. Nonetheless, the 18^#^ sampling point in the point source pollution area had the largest potential ecological risk index (*RI*) of 113.98 among the 19 sampling points, however, the *RI* values of all sampling points were less than 150 that also presented moderate ecological hazards. This largely signified that the HMs in the sediments of the Shahe reservoir were in a tolerable polluted state, except As with moderately polluted stage. The possible sources could be the industrial, domestic, livestock and poultry breeding wastewaters in the Wenyu river basin and the Changping industrial zone, as well as the extensive ambient agricultural production. This mainly include the As-containing pesticides, fertilizers and the herbicides^31^. Therefore, the typical order of prevalent HM pollution is as; As > Cu > Pb > Cr > Zn > Mn. Conversely, it was different from the geo-accumulation index method, principally because of greater Pb toxicity coefficient relative to Cr and Zn, elucidating relatively larger potential ecological hazards for Pb.

**Table 5:** The HM accumulation index, individual ecological risk coefficient and potential ecological risk index in surface sediments.

Sampling site	As		Cr		Cu		Mn		Pb		Zn		*RI*
	*I*_*geo*_	${E}_{r}^{i}$	*I*_*geo*_	${E}_{r}^{i}$	*I*_*geo*_	${E}_{r}^{i}$	*I*_*geo*_	${E}_{r}^{i}$	*I*_*geo*_	${E}_{r}^{i}$	*I*_*geo*_	${E}_{r}^{i}$	
1	*1.90*	***55.85***	0.78	5.14	0.74	12.56	− 0.59	1.00	− 0.30	6.08	0.66	2.38	90.12
2	*1.74*	***50.07***	0.49	4.20	0.53	10.79	− 1.13	0.68	− 0.62	4.87	− 0.08	1.42	78.69
3	*1.38*	38.99	0.59	4.51	0.80	13.05	− 0.36	1.17	− 0.02	7.40	0.59	2.26	75.37
4	*1.55*	***44.01***	− 0.04	2.29	0.19	8.58	− 3.06	0.18	− 1.78	2.18	− 2.37	0.29	61.30
5	*1.75*	***50.40***	0.25	3.56	0.64	11.69	− 2.41	0.28	− 0.79	4.33	− 1.25	0.63	76.12
6	*1.80*	***52.09***	0.39	3.93	0.45	10.25	− 1.31	0.60	− 0.33	5.97	0.52	2.15	82.03
7	*1.62*	***46.17***	0.07	3.14	0.00	7.51	− 1.48	0.54	− 0.85	4.17	*1.66*	4.75	69.99
8	*1.62*	***46.03***	0.31	3.73	0.21	8.70	− 0.90	0.80	− 0.58	5.01	− 0.60	0.99	70.26
9	*1.78*	***51.43***	0.41	3.97	0.60	11.34	− 1.24	0.63	− 0.82	4.26	− 0.12	1.38	79.78
10	**2.02**	***60.69***	0.87	5.48	*1.03*	15.32	− 0.75	0.89	− 0.04	7.31	0.88	2.75	100.70
11	*1.95*	***58.10***	0.62	4.63	0.85	13.55	− 1.05	0.73	− 0.15	6.77	*1.01*	3.01	95.75
12	*1.53*	***43.43***	0.33	3.77	0.54	10.93	− 1.33	0.60	− 0.52	5.23	0.08	1.59	72.12
13	*1.53*	***43.43***	− 0.41	2.26	− 0.79	4.34	− 1.48	0.54	− 1.23	3.19	− 1.49	0.53	56.91
14	*1.73*	***49.72***	0.43	4.04	0.69	12.08	− 0.97	0.77	− 0.34	5.93	0.80	2.61	81.82
15	*1.53*	***43.35***	0.17	3.38	− 0.21	6.47	− 1.78	0.44	− 1.07	3.57	− 0.15	1.35	62.26
16	*1.74*	***50.14***	0.39	3.94	0.37	9.71	− 1.55	0.51	− 0.60	4.95	0.07	1.58	76.67
17	*1.71*	***48.99***	0.50	4.23	0.32	9.35	− 1.97	0.38	− 2.06	1.80	*1.16*	3.35	74.19
18	**2.34**	***75.86***	0.43	4.03	*1.20*	17.20	− 1.76	0.44	− 0.18	6.64	*1.69*	4.85	113.98
19	*1.71*	***49.23***	0.22	3.48	0.80	13.09	− 1.51	0.53	− 0.54	5.14	*1.31*	3.71	79.31
Mean	*1.73*	***50.42***	0.36	3.91	0.47	10.87	− 1.40	0.62	− 0.67	4.99	0.23	2.19	78.81

The bold font indicates; when *I*_*geo*_ is 2–3, moderate pollution; italic font indicates; when *I*_*geo*_ is 1–2, near moderate pollution and; the bolditalic font indicates; the ${E}_{r}^{i}$ in 40–80, moderate ecological hazard.

**Figure 4:**
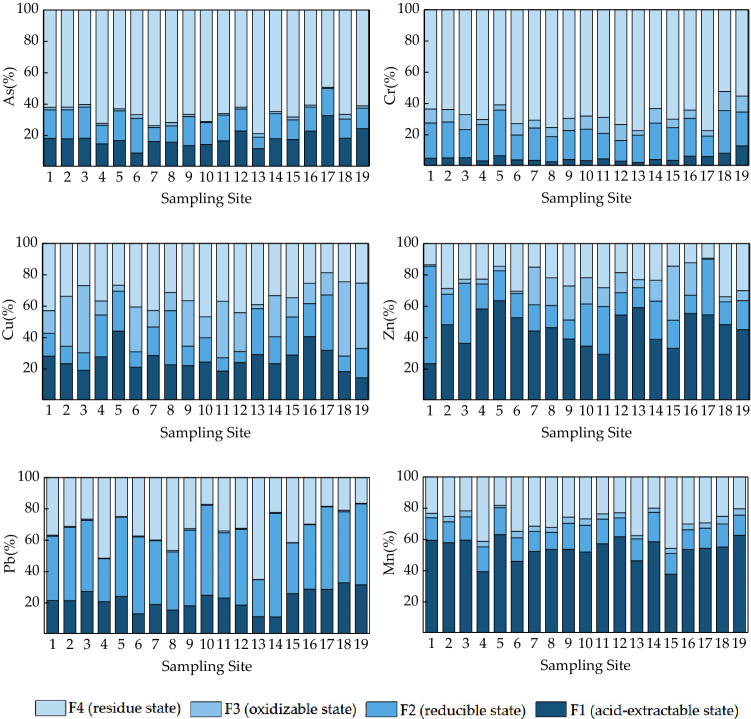
The percent content of various speciation of HMs in surface sediments relative to the total count.

The morphological distribution of As, Cr, Cu, Mn, Pb, and Zn in the surface sediments of the Shahe reservoir has presented in Fig. 4, exhibiting significant variations in the morphological distribution of different HMs. Among them, As and Cr were mainly existed in the residual state with an average proportion of 66% and 68% in all 19 sampling points. In addition, a relatively uniform distribution of Cu was examined in each form, yet it’s residual form characteristically accounted for major fraction. It should be noted that the average proportion of ions was 34%, with the acid-extractable, reducible, and oxidizable states accounting for 27%, 18%, and 21%, respectively. In particular, both Mn and Zn mainly identified in acid-extractable state, with an average proportion of 55% and 45%, respectively, whereas, Pb was distinguished in reducible form, accounting for 45% of the total proportion. It was believed that different HM embraced diverse bioavailability and migration capacity^32^. Among the four forms obtained by BCR continuous extraction, the acid-extracted state has the strongest bioavailability, followed by the reducible and oxidizable states, with the residual state specified as the weakest^33^. In general, the acid-extracted HMs are easily released under acidic or neutral conditions, and are absorbed by plants through migration and transformation mechanisms, and elicit highest bioavailability and toxicological consequences^34^. On the other hand, the reducible state is comparably more stable than the acid-extracted state, however, it can be readily released under reduced water redox potential or anoxic water incidents, which is a compatible for systematic plant uptake^35^; alternatively, the oxidizable state is a conventionally stable and can only be released under strong oxidizing conditions, which tangles plant efficient uptake. Lastly, the residual state often bound in the lattice of primary and secondary silicate minerals. Thus, it is not bioavailable for ambient plants^36^. Table 6 illustrates the RAC values for each studied HM calculated by the risk assessment coding method. Among them, the RAC values of As, Cu, and Pb ranged 10–30% explicating medium risk, implying lower bioavailability. Moreover, 30–50% RAC level of Zn indicated higher risk, with strong bioavailability and competent plant uptake. At most sampling points however, the recorded RAC value of Mn was greater than 50% inferring strong bioavailability with extremely higher risk level.

**Table 6:** RAC value of HMs in surface sediments.

Sampling site	RAC					
	As	Cr	Cu	Mn	Pb	Zn
1	***18.20%***	5.05%	***28.25%***	**59.49%**	***21.50%***	***23.50%***
2	***17.93%***	5.25%	***23.44%***	**57.98%**	***21.49%***	*48.24%*
3	***18.24%***	5.38%	***19.14%***	**59.61%**	***27.32%***	*36.40%*
4	***14.69%***	3.37%	***27.78%***	*39.47%*	***20.80%***	**58.35%**
5	***16.76%***	6.62%	*44.21%*	**63.12%**	***24.19%***	**63.60%**
6	8.90%	4.07%	***21.14%***	*46.08%*	***13.09%***	**52.75%**
7	***16.25%***	3.71%	***28.61%***	**52.39%**	***18.99%***	*44.32%*
8	***15.74%***	2.81%	***22.73%***	**53.69%**	***15.45%***	*46.51%*
9	***13.60%***	4.27%	***22.19%***	**53.74%**	***18.18%***	*39.20%*
10	***14.33%***	3.53%	***24.55%***	**52.06%**	***24.59%***	*34.68%*
11	***16.63%***	4.66%	***18.80%***	**57.26%**	***23.10%***	***29.42%***
12	***22.87%***	3.18%	***24.27%***	**61.79%**	***18.63%***	**54.51%**
13	***11.69%***	2.29%	***29.33%***	*46.43%*	***11.23%***	**59.20%**
14	***18.02%***	4.22%	***23.49%***	**58.60%**	***11.03%***	*38.98%*
15	***17.51%***	3.66%	***28.97%***	*37.86%*	***25.92%***	*33.29%*
16	***22.81%***	6.42%	*40.63%*	**53.61%**	***28.76%***	**55.52%**
17	*32.67%*	6.21%	*31.94%*	**54.39%**	***28.69%***	**54.56%**
18	***18.25%***	8.29%	***18.34%***	**55.18%**	*32.89%*	*48.36%*
19	***24.37%***	***13.04%***	***14.45%***	**62.70%**	*31.53%*	*45.16%*
Mean	***17.87%***	5.05%	***25.91%***	**53.97%**	***21.99%***	*45.61%*

The bold font indicates: RAC > 50%, very high risk; italic indicates: RAC is 30–50%, high risk; bolditalic indicates: RAC is 10–30%, medium risk.

### Source analysis of HMs in sediments

**Figure 5:**
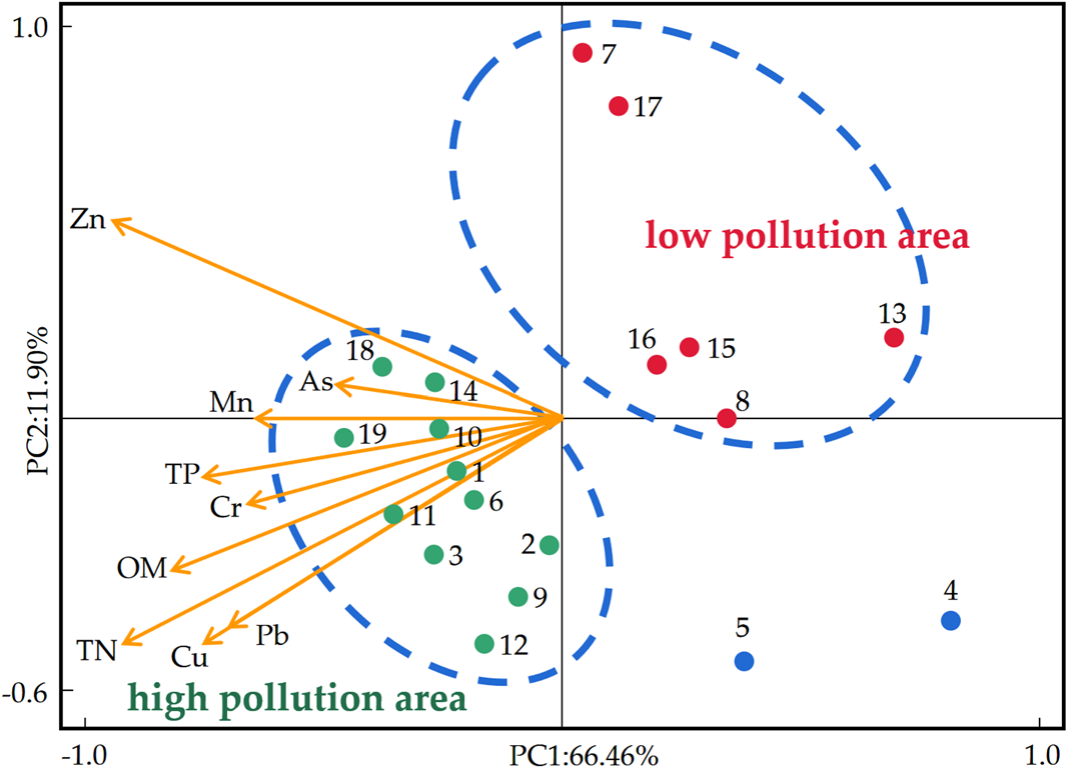
Principal component analysis of HMs with N and P pollutants in sediments.

Combined with the spatial distribution characteristics of nutrients and organic matter in the sediments of Shahe reservoir was analyzed by a pervious method with minor modifications^16^ pearson correlation analysis and PCA were performed on the contents of heavy metals, total nitrogen (TN), total phosphorus (TP) and OM in the sediments of Shahe reservoir. As shown in Table 7, except As and TP, a significant correlation was found between As and Zn as well as As and organic matter (OM) (*p* < 0.05). This observation suggested that most of these studied HMs had potentially similar sources and migration pathways. An earlier study documented that higher loads of Cd, Pb, Cr, Zn, Cu and other HMs in wastewater comes from mining, metallurgy, electroplating and other industries^37^. The As streams are linked with heavy agricultural application of pesticides, and the automobile exhaust exclusively liable for Pb pollution, while Mn could be associated battery production, etc. Altogether, these sources substantially enrich various downstream water bodies with HM contamination. As expected, most of the HMs in the sediments of the Shahe reservoir showed a significant correlation with nitrogen (N), phosphorus (P) and OM. Previous studies have conducted N and P traceability analysis in the Shahe reservoir, and it was believed that the pollution may come from both organic Nand P components. In addition, the particulate N and P found to be adsorbed on organic matter components, suggesting that point source pollution could be an important source of OM in the sediments of the Shahe reservoir^16^. Additionally, the PCA findings revealed that 11 sampling points credited severe pollution zones due to diverse polluting factors (Fig. 5). Specifically, these zones include sampling points 1^#^ and 2^#^ in the upstream, and sampling points 3^#^, 9^#^, in the center of the reservoir. Besides, the sampling points 10^#^ and 11^#^, sampling points 6^#^ and 12^#^ in the downstream of the reservoir, points 18^#^ and 19^#^ in the point source pollution area, and sampling points 14^#^ in the Beisha river channel located in the downstream of the point source pollution area. Similarly, the main polluted areas of the Shahe reservoir were concentrated in the central and downstream zone and the point source pollution areas.

**Table 7:** Correlation analysis of pollutants in sediments.

	Cr	Mn	Cu	Zn	As	Pb	TN	TP	OM
Cr	1	0.694**	0.716**	0.512**	0.505**	0.671**	0.585**	0.364**	0.362**
Mn		1	0.566**	0.404**	0.355**	0.787**	0.541**	0.518**	0.425**
Cu			1	0.818**	0.503**	0.787**	0.658**	0.527**	0.488**
Zn				1	0.301*	0.682**	0.479**	0.487**	0.362**
As					1	0.410**	0.380**	0.110	0.286*
Pb						1	0.653**	0.588**	0.513**
TN							1	0.662**	0.792**
TP								1	0.585**
OM									1

**p < 0.01; *p < 0.05.

## Conclusion

1. The order of enrichment multiples of heavy metals in different areas of the Shahe Reservoir compared with the soil background value is: point source pollution area > reservoir center > lower reservoir > Nansha River > upper reservoir > Beisha River. The columnar samples in the reservoir center and the Beisha River inlet showed a trend of first increasing and then decreasing with the change of depth, and the overall pollution was more serious at the depth of 25–30 cm.
2. The pollution evaluation of 6 heavy metals showed that except As, which was more than moderate pollution, the other heavy metals had no pollution or light pollution, and the RI values of all sampling points were less than 150, which belonged to mild ecological hazards. In addition, the heavy metal forms of As, Cr, and Cu are mostly residues with low bioavailability, while Mn and Zn are not seriously polluted, but have strong bioavailability.
3. The distribution of heavy metals, total nitrogen, total phosphorus and organic matter content in the sediments of the Shahe Reservoir, except As and total phosphorus, all showed a significant correlation between the two, with potentially similar sources and migration routes, mainly mixed. There are industrial pollution, surface runoff pollution and point source pollution. And the results of principal component analysis show that the high pollution areas are mostly concentrated in the center of the reservoir, downstream of the reservoir and point source pollution areas.

## Data Availability

The datasets used and/or analysed during the current study available from the corresponding author on reasonable request.

## References

[1] Xu XW, Liu DW, Che HJ (2009). Investigation and evaluation of water environment in the North Canal. Haihe Water Conserv..

[2] Facchinelli A, Sacchi E, Mallen LJ (2001). Multivariate statistical and GIS-based approach to identify heavy metal sources in soils. Environ. Pollut..

[3] Chen J, Wang J, Guo J (2018). Eco-environment of reservoirs in China. Phys. Geogr. Earth Environ..

[4] Ding HJ (2009). Analysis of Heavy Metal Pollution in Sediments in the North Canal Basin.

[5] Liu G, Jiang CY, Lee B (2018). Concentration distribution and risk index of heavy metals in sediments of Chaohu Lake. Environ. Sci. Technol..

[6] Zhang J, Guo X, Zeng Y (2019). Distribution and pollution assessment of heavy metals in river sediments in Taihu Lake Basin. Environ. Sci..

[7] Pan LB, Wu RH, Wang L (2019). Heavy metal pollution degree and risk assessment of soil and sediment in the upper reaches of Miyun Reservoir in Beijing. J. Environ. Eng. Technol..

[8] Shi HH, Jiao CC, Zengetc. J (2019). Vertical distribution and pollution assessment of heavy metals in sediments of Dianchi Lake. People's Yangtze River.

[9] Jing HW, Zhang ZG, Guo J (2013). Analysis of water pollution characteristics and pollution sources of Beijing North Canal water system. Chin. Environ. Sci..

[10] Li LF, Zeng XB, Li GX (2007). Risk assessment of heavy metal pollution in sediments of Wenyu River in Beijing. J. Environ. Sci..

[11] Shan BQ, Jian YX, Zhang H (2011). Characteristics and evaluation of heavy metal pollution in sediments in the lower reaches of the North Canal. J. Saf. Environ..

[12] Zhang W, Zhang H, Shan BQ (2012). Study on the characteristics of heavy metal pollution in sediments of Shahe Reservoir in the headwaters of the North Canal. Environ. Sci..

[13] Xu, M. *Research on Ecological Impact Assessment of the North Canal and Joint Dispatch of Water Quality and Quantity* (North China Institute of Water Conservancy and Hydropower, Zhengzhou, 2010)

[14] Yuan SG, Zhang WJ, Zheng B (2014). Distribution characteristics and diffusion flux estimation of heavy metals in sediments of Shahe Reservoir in Beijing. J. Saf. Environ..

[15] Zhou, X. Y., Zhang, J. H., Qu, D., etc. Analysis on the pollution characteristics of rainwater runoff in the surrounding ditches of Shahe Reservoir in Beijing. In *Chongqing: The 13th China Urban Water Development International Symposium and New Technology Equipment Expo* (2018).

[16] Sun W, Wang LM, Liu JB (2019). Characteristics and traceability analysis of nutrient distribution in sediments of Shahe Reservoir in North Canal. J. Environ. Sci..

[17] Tessier A, Campbell PGC, Bission M (1979). Sequential extraction procedure for the speciation of particulate trace metals. Anal. Chem..

[18] Yang H, Zhang YG (2014). Determination of 6 heavy metal elements in water system sediments by inductively coupled plasma atomic emission spectrometry (ICP-OES). China Inorg. Anal. Chem..

[19] Pardo, R., Helena, B. A., Cazurro, C. et al. Application of two- and three-way principal component analysis to the interpretation of chemical fractionation results obtained by the use of the BCR procedure. *Anal. Chimica Acta* **523**(1), 125–132 (2004)

[20] Xiao WS, Yang K, Guo JL (2008). Heavy metal pollution and potential ecological risk assessment in the sediment of Cihu Lake. Environ. Eng..

[21] Gao YX, Feng JG, Tang L (2012). Species distribution and risk assessment of heavy metals in the soil of metal mining areas in the upper reaches of Miyun Reservoir. Environ. Sci..

[22] Hakanson L (1980). An ecological risk index for aquatic pollution control. A sedimentological approach. Water Res..

[23] Yu Y, Gao HC, Maetc. JH (2013). Analysis and evaluation of soil heavy metals in Chaohe Basin in Miyun County. Environ. Sci..

[24] Wang JK, Zeng XL, Xu DY (2020). Chemical fractions diffusion flux and risk assessment of potentially toxic elements in sediments of Baiyangdian Lake, China. Sci. Total Environ..

[25] Hu XL, Zhao LX, Liao RH (2011). Evaluation of heavy metal pollution in Wenyu River sediments. Beijing Water.

[26] Yu DW, Yu M, Weietc. YS (2012). Temporal and spatial evolution characteristics of water environment quality of Wenyu River from 1980 to 2010. J. Environ. Sci..

[27] Jiao HL (2019). Heavy metals in urban domestic sewage and sludge. Chem. Eng. Equip..

[28] Guo J, Ma L, Shi XY (2011). Monitoring and analysis of rainfall and runoff on urban roads in Beijing. Environ. Chem..

[29] Hou PQ, Ren YF, Wang XK (2012). Research on water quality evaluation of urban rainfall runoff in Beijing. Environ. Sci..

[30] Chang HD, Jin PK, Fu BW (2016). Sediment properties of drainage pipes in different functional areas of Kunming City. Environ. Sci..

[31] Yu XJ, Huo SL, Zan FY (2012). Distribution characteristics and pollution assessment of arsenic in the surface sediments of Chaohu Lake. J. Environ. Eng. Technol..

[32] Jiang SX, Zhai FJ, Zhang C (2020). Distribution characteristics and risk assessment of heavy metals in sediments of Yitong River (urban section). Environ. Sci..

[33] Jin GQ, Fu LQ, Huang QY, etc. (2019). Analysis on the occurrence form and bioavailability of heavy metals in farmland soil: A 100-mu farmland in Jinhua City as an example. Green Tech.

[34] Jiang L, Gong XF, Yuanetc. SF (2020). Study on the quality benchmark of heavy metals in sediments of Poyang Lake and its ecological risk assessment. Environ. Pollut. Prevent..

[35] Gao X, Chen CT (2012). Heavy metal pollution status in surface sediments of the coastal Bohai Bay. Water Res..

[36] Bao XM, Chao JY, Yin HB (2016). Occurrence characteristics and bioavailability of heavy metals in the sediment of Gehu Lake in Taihu Lake Basin. Lake Sci..

[37] Zhang C, Tan XJ, Wang L (2019). Study on heavy metal content and source traceability of urban sludge in key river basins in my country. Water Drain..

